# Blends Based on Poly(ε-Caprolactone) with Addition of Poly(Lactic Acid) and Coconut Fibers: Thermal Analysis, Ageing Behavior and Application for Embossing Process

**DOI:** 10.3390/polym14091792

**Published:** 2022-04-27

**Authors:** Dino Priselac, Sanja Mahović Poljaček, Tamara Tomašegović, Mirela Leskovac

**Affiliations:** 1 Faculty of Graphic Arts, University of Zagreb, 10000 Zagreb, Croatia; 2 Faculty of Chemical Engineering and Technology, University of Zagreb, 10000 Zagreb, Croatia; mlesko@fkit.hr

**Keywords:** poly(ɛ-caprolactone), poly(lactic acid), coconut fibers, blends, printing plate, embossing process

## Abstract

In this research a biodegradable blend of poly(ɛ-caprolactone) (PCL) and poly(lactic acid) (PLA) is proposed as a new material for the production of a relief printing plate used for special applications on packaging materials, i.e., the embossing process. Coconut fibers (CFs) were added as a natural filler to PCL/PLA blends to improve the functional properties of the prepared blends. Thermal, mechanical and surface analyses were performed on the unaged and artificially aged blends. The results showed that CF has been proven to optimize the hardness of the blend, which is crucial for the production of relief plate for embossing applications. The lowest hardness was measured on neat PCL (53.30° Sh D) and the highest value on PCL/PLA/CF 70/30/3.0 blend (60.13° Sh D). Stronger interfacial interactions were present at the PLA/CF interface because the interfacial free energy is closer to zero and the work of adhesion and spreading coefficient are higher than for the PCL/CF interface. The results of thermal analysis of unaged and aged blends showed that ageing for 3 weeks resulted in significantly lower thermal stability, especially for neat PCL and PCL/PLA 80/20 blends. Blends with a higher content of PLA and CF showed a slightly increased ageing resistance, which is attributed to the increased crystallinity of PLA after ageing due to the addition of CF showed in the DSC diagrams.

## 1. Introduction

Due to the increasing awareness of the environmental hazards caused by the disposal of conventional polymeric materials, studies directed towards the production and use of biodegradable polymers have become more widespread. In recent decades, therefore, there has been a great interest in the development of polymeric materials that can replace the traditional fossil-based materials and have significant environmental benefits. They can currently be found in the packaging industry, agriculture, medicine and other fields such as electronics, the automotive industry and construction [1,2]. The most popular are aliphatic polyesters such as polylactide (PLA), polycaprolactone (PCL), polyethylene oxide (PEO), poly(3-hydroxybutyrate) (PHB), polyglycolide (PGA) and poly(lactide-*co*-glycolide) (PLGA) [2,3,4]. Other bio-based materials such as biophenolic resin are also widely used [5].The advantage of biodegradable materials over polymeric materials derived from petroleum sources is their ability to degrade in a much shorter time than conventional polymers [6,7]. However, despite their advantages, they also have some limitations in their properties and price. They are still quite expensive compared to conventional polymers, and this is definitely one of the most serious limitations that restricts their application [8]. Some natural polymers are available in large quantities and have a lower price, but their properties are often further from those of conventional plastics. To overcome the limitations in terms of their properties, different approaches can be used to obtain a composite matrix with adapted properties [2]. In this context, the modification of different types of biodegradable materials by mixing and compatibilization processes is one of the most interesting topics within materials research [8,9,10,11,12,13,14,15,16].

One of the most interesting biodegradable polymers that can be used for a wide range of applications is PLA. PLA is a thermoplastic aliphatic polyester derived from natural sources such as corn, sugar beets and tapioca roots [3,17]. It is mainly used in packaging, agriculture and clothing, in 3D printing application as a filament and for medical purposes [18,19,20,21]. PLA is stiff and brittle below its glass transition temperature (*T_g_*), which is in the range of 50 to 60 °C [22]. In certain applications, the major weakness of PLA is its stiffness and brittleness. To overcome these limitations, PLA is often blended with a nanoscale filler or a second polymer to improve its properties [3,8,14,23,24]. For example, silicon dioxide (SiO_2_) nanoparticles have been incorporated into PLA composite to improve its mechanical properties, fire resistance and thermal stability [25]. In another study, the effects of adding modified silica nanoparticles on the thermal and mechanical properties of PLA were reported [26]. The authors concluded that the surface area difference of silica plays an important role in modifying the polarity of nanoparticles and their integration and properties of partially polar polymers. Furthermore, in order to make immiscible blends of PLA compatible with low-density polyethylene (LDPE), nano-calcium carbonate (CaCO_3_) and silica nanofillers were used. Research published on this topic has shown that the addition of silica to PLA/LDPE blends can improve the mechanical properties of the observed blend [27]. One of the biodegradable polymers commonly used in preparation of PLA blends is PCL. PCL has excellent deformability, low glass transition temperature, *T_g_* = −60 °C and its melting temperature is *T_m_* = 60–65 °C [1]. One of the most interesting properties of PCL is relatively good miscibility and compatibility with other polymers [28,29]. In prepared polymer blends, the addition of PCL can improve the resistance to cracking due to its good elastic properties. The studies published on this topic showed that blending PLA and PCL leads to a decrease in the stiffness and strength of produced blends with a limited improvement in toughness [2,23,24,30,31]. The conclusions revealed that these results are a consequence of weak interfacial adhesion and low ability of mixing of these components. Considering that PLA and PCL are immiscible polymers, the addition of compatibilizers to PLA/PCL blends can increase their miscibility and thus improve the properties of the resulting blends. PLA/PCL blends with the addition of a third component that plays the role of a compatibilizer have been reported and show a wide range of physical properties and biodegradability that can be controlled by adjusting the proportion of components and blending conditions [8,9,10,11,12,13,14,15,32].

**Figure 1:**
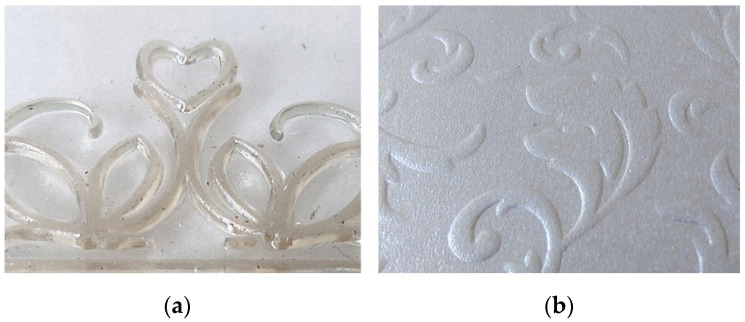
Segment of the (**a**) printing plate and (**b**) embossed image.

Considering the increasing use of biodegradable materials in many industries to reduce the negative impact of conventional plastics, the need to switch to environmentally friendly processes and materials has also arisen in the printing industry [33,34]. Apart from the aforementioned use of biodegradable materials in the packaging industry, where biodegradable polymers are mainly used as printing substrates, another segment of printing technology is interesting because of the possibility of replacing the conventional polymers with biodegradable alternatives. This is applicable to the production of printing plates, materials that are considered an integral part of the printing process as they are used to transfer printing inks/designs to the substrate. One of the methods of transferring designs from the printing plate to the substrate is embossing. Embossing is a type of printing process that creates an embossed image of a design on various packaging materials and other substrates. Therefore, the printing plates used in embossing have raised image elements and recessed non-image elements and are usually made of metals and/or various types of fossil-based polymers (Figure 1). Previously published studies on embossing printing plates have shown that a blend of PCL and PLA with a coconut fiber has significant potential to produce materials that match the properties of conventional polymeric materials currently used as relief printing plates [35,36,37].

In this study, a further analysis was conducted on unaged and artificially aged three-component blend containing PCL, PLA and coconut fibers (CFs). Coconut fiber can be added as a reinforcement in the PCL/PLA blends to adjust their properties to the qualitative requirements of the printing plate for embossing. It is a natural fiber obtained from coconut bark. It is composed of cellulose, hemicellulose and lignin; the fibers are thin and hollow with thick cellulose walls [38]. Coconut fiber is used to make of various products such as carpets, brushes and ropes and is also used in agriculture [39]. It is a very versatile material that can be used in various technical fields as a sustainable building material [40].

The aim of the present study was to analyze the thermal as well as important surface properties of the unaged and artificially aged PCL/PLA/CF blends. Furthermore, when choosing the materials and preparing polymer blends for this research, specific attention was dedicated to obtaining the blend with Shore D hardness in the range of the hardness of commercial materials used for embossing printing plates. The influence of artificial ageing of the prepared blends was observed to evaluate the stability and possible degradation process of PCL/PLA/CF blends.

## 2. Materials and Methods

### 2.1. Materials

PCL was supplied by Capa 6800, Perstorp, Warrington, UK, while PLA was supplied by InegoTM 3251D, Nature Works LLC, Plymouth, MN, USA. The polymer matrix for the preparation of the blends was made of PCL, while PLA and coconut fibers were added at a certain ratio. The properties of the PCL and PLA used are listed in Table 1.

### 2.2. Preparation of Biodegradable Blends

PCL and PLA with addition of CF were blended in the Brabender^®^ internal mixer at a temperature of 190 °C. The kneader speed was 60 rpm. The samples were mixed for 5 min, after which they were cut into pieces. Then, the cut pieces were molded into plates with dimensions of 100 mm × 100 mm × 1 mm using a hydraulic press at a temperature of 190 °C and a pressure of 16 MPa. The pressing process took seven minutes: two minutes of preheating and five minutes of pressing. Table 2 shows the composition of PCL/PLA/CF blends.

### 2.3. Ageing Process

In this study, the ageing process was performed in the laboratory by exposing the samples to a xenon lamp to simulate sunlight UV in a test chamber Solarbox 1500e (CO.FO.ME.GRA., Milano, Italy). The xenon light exposure simulates realistic natural outdoor weathering conditions. In this research, a S208/S408 (artificial daylight) indoor filter was used to simulate indoor weathering with daylight and had an infrared reflective coating to prevent overheating of the samples during exposure. Irradiation was set at 550 W/m^2^ at a temperature of 50 °C. The equipment was set according to the standard ISO 4892-2. All the samples were exposed for different periods of time: one, two and three weeks in total. 

### 2.4. Thermal Analysis

#### 2.4.1. Differential Scanning Calorimetry Analysis

The thermal properties of neat PCL and two and three component blends were analyzed using differential scanning calorimetry (DSC) on a Mettler Toledo DSC 823e, US. Approximately 10 mg of each sample was cut from the plates and placed in an aluminum pan. Tests were performed at a flow rate of 50 cm^3^·min^−1^ under an inert nitrogen atmosphere and cooled by an Intracooler at a heating/cooling rate of 10 °C/min. Measurements were performed during two heating cycles and one cooling cycle in the temperature range from −90 to 200 °C. The first heating cycle (up to 200 °C) was performed to eliminate thermal history of the sample preparation. The samples were then cooled to −90 °C and heated to 200 °C in the second cycle. Samples were analyzed in the first cooling and the second heating cycle. 

The glass transition temperature (*T_g_*), melting point (*T_m_*) and crystallization temperature (*T_cc_*) were determined as well as the cold crystallization enthalpy (Δ*H_cc_*) and the melting enthalpy of the samples *(*Δ*H_m_*).

Based on the determined enthalpy of fusion of the PCL samples, the percentage of crystallinity (*X*_*c*(PCL)_) was calculated according to Equation (1) [41]:

(1) $$X_{c\left(\mathrm{PCL}\right)}=\frac{\Delta H_{m\;}}{\Delta H^{0}{}_{m\;}\times w_{\;}}\times 100$$

where *X*_*c*(PCL)_ is the percentage of crystallinity of PCL; Δ*H_m_* is the specific enthalpy of fusion [J·g^−1^]; Δ*H*^0^*_m_* is the enthalpy of fusion of 100% crystalline polymer (where the enthalpy of fusion of 100% PCL is 139.3 J·g^−1^ [28]) and *w* is the mass fraction of PCL.

The fraction of PLA crystallinity (*X*_*c*(PCA)_) was calculated from the enthalpy of fusion and cold crystallization according to Equation (2):

(2) $$X_{c\left(\mathrm{PLA}\right)}=\frac{\left(\Delta H_{m\;}-\Delta H_{cc\;}\right)}{\Delta H^{0}{}_{m\;}\times w_{\;}}\times 100$$

where *X*_*c*(PCA)_ is the percentage of crystallinity of PLA; Δ*H_m_* and Δ*H_cc_* are the enthalpy of fusion and cold crystallization of PLA [J·g^−1^]; Δ*H*^0^*_m_* is the enthalpy of fusion of 100% crystalline PLA (where the enthalpy of fusion of 100% PLA is 106 J·g^−1^ [42]) and *w* is the mass fraction of PLA.

#### 2.4.2. Thermogravimetric Analysis

The thermal stability of polymer blends was determined by thermogravimetric analysis (TGA) on a TA Instruments Q500 instrument, New Castle, DE, USA. Thermogravimetric analysis measures the change in sample mass during the temperature change. In this study, the temperature range was between 25 and 900 °C. The mass of the sample placed in the thermobalance was about 10 mg. The samples were heated in an open platinum crucible in an inert atmosphere of nitrogen at a flow rate of 60 cm^3^/min.

### 2.5. Mechanical Measurements

The hardness of the samples was measured using the Shore D method, which is most commonly used in measuring the hardness of hard plastics and tires. For this measurement, the Zwick Roell 3130 Hardness Tester, Ulm, Germany, was used, which operates according to the standard ISO 7619-1. The procedure is carried out by placing a sample of several layers of the polymer (4 mm high) into device. The steel needle is lowered onto the polymer material, and the hardness value is displayed on the digital screen. The hardness was expressed in ° Sh D as a mean value of 10 measurements.

### 2.6. Surface and Interfacial Characteristics

#### 2.6.1. Surface Analysis

To determine the changes in the surface properties of the prepared blends, the surface free energy (SFE) of the samples was calculated. To determine the SFE of solids (γ_s_), the contact angles (θ) of probe liquids with known polar (γ_l_*^P^*) and dispersive (γ_l_*^D^*) components should be measured [43]. Water, glycerol and diiodomethane were used for calculation of SFE. The conductivity of water was γ = 2,0 μS·cm^−1^, the dispersive component of the surface tension was γ_l_*^D^* = 21.80 mJ·m^−2^, the polar component of the surface tension was γ_l_*^P^* = 51.00 mJ·m^−2^ and the total surface tension of the water was γ_l_ = 72.80 mJ·m^−2^. Glycerol has an equal amount of both components (γ_l_*^P^* = 34.0 mJ·m^−2^, γ_l_*^D^* = 30.0 mJ·m^−2^) and total surface tension γ_l_ = 64.0 mJ·m^−2^, and diiodomethane is a dispersive liquid with total surface tension, γ_l_ = γ_l_*^D^* = 50.8 mJ·m^−2^ [43]. For contact angle measurement, a sessile drop method was used. The shape of the droplets was a spherical cap, and the volume of the drops was 1 µL. All measurements of the contact angle on the samples were performed at the same moment after the droplet touched the sample surface, and the average value of 10 measurements was calculated. The measurements were performed by means of a Goniometer DataPhysics OCA 30 (DataPhysics Instruments GmbH, Filderstadt, Germany). The results of the contact angle measurements and the surface tension of the probe liquids were used to calculate the SFE of the samples. 

In order to obtain information about the strength of interactions between the blended materials, the adhesion parameters were calculated by using the values of surface free energies of neat PCL, PLA and CF in form of the pressed pellets. Interfacial free energy (γ*_mf_*), work of adhesion (*W_mf_*) and spreading coefficient (*S_mf_*) were calculated using Equations (3)–(5) where subscripts *A* (matrix) and *B* (filler) mean phases in composites and superscripts *d* and *p* mean dispersed and polar components [44]. 

SFE of the interphase (γ*_AB_*) was calculated according to Equation (3):

(3) $$\gamma_{AB}=\gamma_{A\;}+{\;\gamma }_{B\;}-2\;\sqrt{\gamma_{A}^{D}\;}\gamma_{B}^{D}-2\;\sqrt{\gamma_{A}^{P}\;}\gamma_{B}^{P}$$

The work of adhesion (*W_AB_*) between the materials was calculated according to Equation (4):

(4) $$W_{AB}=\gamma_{A}+\gamma_{B}-\gamma_{AB}$$

The spreading coefficient (*S_AB_*) was calculated according to Equation (5):

(5) $$S_{AB}=\gamma_{A}-\gamma_{B}-{\;\gamma }_{AB}$$

#### 2.6.2. Fourier Transform Infrared Spectroscopy

Fourier transform infrared spectroscopy (FTIR-ATR) was used to detect the changes in the structure of the polymer blends. FTIR-ATR analysis was performed at ambient temperature using a Perkin Elmer Spectrum One spectrometer, Waltham, MA, US. Spectra in attenuated total reflection (FTIR-ATR) mode were recorded in the frequency region of 400–4000 cm^−1^ using a Diamond ATR system with a ZnSe lens. The spectra were recorded with a resolution of 2 cm^−1^ by coadding the results of 10 scans.

#### 2.6.3. Image Analysis of Engraved Printing Plates

In order to evaluate the potential application of prepared blends for the production of printing plates, the samples were engraved using CO_2_ lasers. CO_2_ lasers are commonly used to design and engrave printing plates made of elastomeric rubber. In the engraving process, a laser removes the part of the material from the samples to form a relief in the printing plate [37,45]. The laser engraving conditions were set the same for each engraved sample. Two- and three-dimensional (2D and 3D) analyses of the engraved samples were performed using a 3D scanning microscope, Troika Systems Limited, Highworth, UK. Flexoplate QC software was used in order to obtain 2D and 3D readings of engraved relief depths.

## 3. Results and Discussion

### 3.1. Thermal Properties

**Figure 2:**
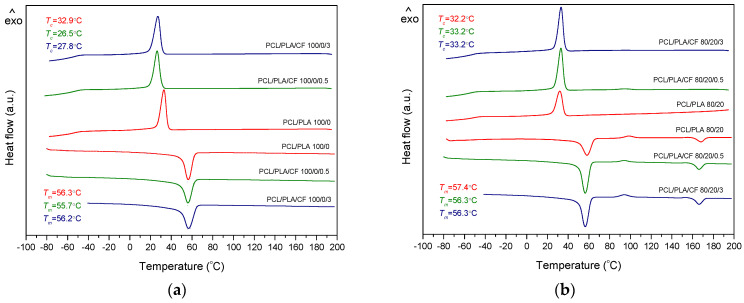

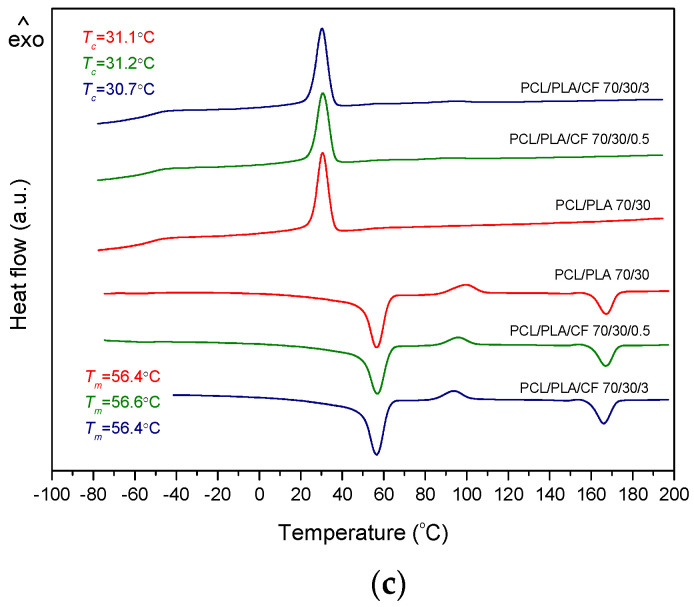
DSC thermograms of PCL/PLA/CF blends during the cooling and second heating cycle: (**a**) 100/0/0, 100/0/0.5, 100/0/3.0; (**b**) 80/20/0, 80/20/0.5, 80/20/3.0 and (**c**) 70/30/0, 70/30/0.5, 70/30/3.0.

Thermal properties of the PCL blends were studied with differential scanning calorimetry (DSC) measurements. DSC analysis was performed to determine the crystallization and melting behavior of the PCL blends and their degradation caused by UV exposure. The obtained second heating and cooling curves of PCL modified with PLA and coconut fibers are shown in Figure 2. The thermal transitions, melting temperature (*T_m_*), melting enthalpy (Δ*H_m_*), temperature of cold crystallization (*T_cc_*), enthalpy of cold crystallization (Δ*H_cc_*), crystallization temperature (*T_c_*) and enthalpy of crystallization (Δ*H_c_*) of neat polymers and polymer blends are listed in Table 3.

According to the results shown in Figure 2 and presented in Table 3, all samples have distinct melting (*T_m_*) and crystallization (*T_c_*) temperatures. The glass transition temperature (*T_g_*) of PCL is around −63 °C, and the *T_g_* of PLA could not be determined because it overlaps with the melting transition of PCL. The corresponding temperatures for neat PCL are 56.3 (*T_m_*) and 32.9 °C (*T_c_*). The melting peak of neat PCL is close to the *T_g_* of neat PLA, which is reported to be around 60 °C, in agreement with the literature [31]. The *T_g_* of PCL and the *T_m_* values for PCL and PLA in blends (about 167 °C) are not strongly affected by the blend composition, indicating their immiscibility [31]. After the addition of natural fibers to PCL, the melting temperatures did not shift significantly. From the results of the percentage of crystallinity (*X_c_*) of PCL with the addition of coconut fibers, it can be seen that no significant change is observed.

From the results presented in Table 3, it can be seen that the addition of PLA to PCL causes the decrease in the enthalpy of crystallization (Δ*H_c_*) and enthalpy of fusion (Δ*H_m_*) of PCL. The addition of PLA and coconut fibers (CFs) to PCL blends does not result in significant changes in melting (*T_m_*) and crystallization (*T_c_*) temperatures. However, it can be seen that the cold crystallization temperature (*T_cc_*) in the PCL/PLA blends is lower when CF is added and that the *T_cc_* of PLA slightly increases by increasing the PLA content in the blend. On the other hand, it can be seen that the addition of PLA polymer has a significant effect on decreasing the degree of crystallinity (*X_c_)* of PCL in PCL/PLA blends, that is, that PLA interferes with the crystallization of PCL. The addition of CF to PCL/PLA blends leads to an increase in the degree of crystallinity of PCL, which may indicate possible interactions with the PLA phase. On the other hand, it can be seen that the degree of crystallinity of PLA in the blends (80/20 and 70/30) increases with the addition of CF. This fact suggests that the presence of natural fibers promotes the crystallization of PCL and PLA. Similar results have been published previously [31,46]. The higher PCL content in the PCL/PLA blends (composition 80/20) contributes to the increase in PLA crystallinity degree, i.e., it induces the crystallization of PLA due to the nucleation effect of the secondary phase. These results are in agreement with the previously published results on the nucleation of PLA in the presence of PCL [47].

**Figure 3:**
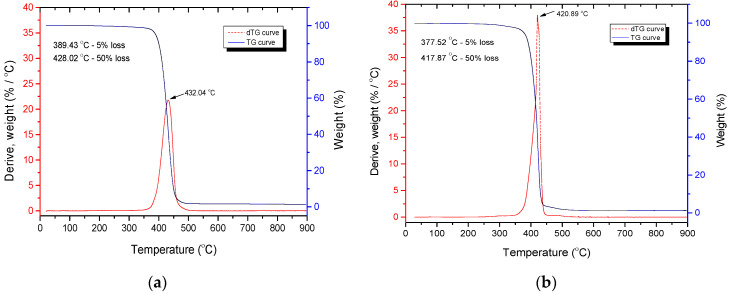
TG and DTG curves of (**a**) neat PCL and (**b**) PCL/PLA/CF 100/0/3.0 blend.

**Figure 4:**
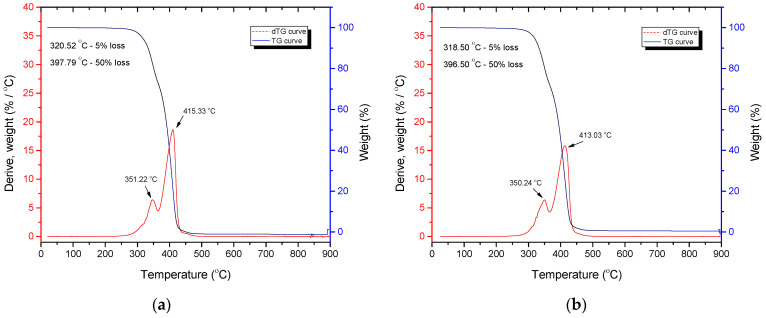
TG and DTG curves of (**a**) PCL/PLA/CF 70/30/0 and (**b**) PCL/PLA/CF 70/30/3.0 blends.

TGA was performed to determine possible changes in thermal stability and thermal degradation rate of polymer blends when different contents of CF were added. TG and DTG curves for selected blends are shown in Figure 3 and Figure 4.

TG and DTG curves shown in Figure 3 and Figure 4 are characteristic of neat PCL and PCL/PLA/CF blends [48]. TG and DTG curves of PCL and PCL/CF composites and PCL/PLA blends presented in Figure 3 exhibit one thermal decomposition step of neat PCL (a) and PCL/CF (b) composite, while Figure 4 exhibits two thermal decomposition steps of PCL/PLA and PCL/PLA/CF blends. The maximum degradation temperature of the DTG curve that corresponds to the highest decomposition rate for PLA is around 351 °C, while for PCL it is around 415 °C. 

However, the addition of CF causes the temperatures of the characteristic points in the thermal degradation process to decrease. Specifically, the onset of degradation (5% weight loss of the sample) shifts from 389.4 °C for neat PCL to 377.5 °C for PCL + 3% CF (Figure 3). The same is true for the PCL/PLA blend: the 5% weight loss occurs at 320.5 °C for the PCL/PLA 70/30 blend without CF and at 318.5 °C for the PCL/PLACF 70/30/3.0 blend (Figure 4). The addition of PLA to PCL is also responsible for the shift to lower temperatures in the degradation process, since these polymers are immiscible, which affects the structure and thermal resistance of the material. The temperature at 50% weight loss also shifts to lower values with the addition of PLA and CF to PCL (Figure 4b). The temperature at maximum degradation rate was shifted from 432.0 °C for neat PCL to 413.0 °C for the PCL/PLA/CF 70/30/3.0 blend. It can be concluded that CF accelerates the thermal degradation of PCL/PLA blends. The results of the TGA show that the addition of PLA and CF decreases the thermal stability of PCL as well as PCL/PLA blends.

### 3.2. Mechanical Measurements

The results of the hardness test of the PCL blends are presented in Table 4. It can be seen that the samples with PLA addition have higher hardness values compared to the neat PCL. The addition of CF increases the hardness values for all the samples. The increase in hardness confirms that the introduction of natural fibers, such as coconut fibers, positively affects the mechanical properties of PCL and PCL blends in terms of usability and adaptation to specific applications as relief printing plates. The hardness of PCL changes from 53.3° Sh D to 54.1° Sh D and 54.4° Sh D with the addition of fibers in small content of 0.5 and 3.0 wt%, respectively. A similar increase in hardness is observed in other samples. The highest value of hardness was measured for the PCL/PLA/CF 70/30/3.0 sample (60.13° Sh D). This confirms the fact that a higher content of PLA and coconut fibers forms a blend with better mechanical properties of neat material (PCL), since the increased hardness values of the printing plate are desirable for the embossing process. These results were expected based on the previously published results where the different formulations of PLA and PCL showed improved mechanical performance and indicated the possibility of adjusting the properties of the blends according to the required functional properties of the material produced [22,31,49,50].

### 3.3. Interfacial Interactions in PCL/PLA/CF Blends

Table 5 shows the calculated total, polar and dispersive surface free energy (SFE) of neat PCL, PLA and coconut fibers (CFs) using the OWRK method [51]. Adhesion parameters such as interfacial free energy (*γ_mf_*), work of adhesion (*W_mf_*) and spreading coefficient (*S_mf_*) between components in polymer blends were calculated and presented [44,52]. 

For the optimal adhesion, interfacial free energy (*γ_mf_*) should be minimal and close to zero, work of adhesion (*W_mf_*) should be maximal and spreading coefficient (*S_mf_*) should be positive [48]. Based on the obtained adhesion parameters, the wetting is optimal between PLA and CF. Moreover, negative spreading coefficient between PCL and PLA is in accordance with their immiscibility [53,54]. Generally, calculated adhesion parameters suggest that CFs are incorporated in PLA matrix, rather than on the interface of PCL and PLA. Specifically, stronger interactions are present at the PLA/CF interface, since interfacial free energy is closer to zero, and work of adhesion and spreading coefficient are higher than for PCL/CF interface. 

### 3.4. Properties of UV Aged PCL Blends

#### 3.4.1. Thermal Properties of UV Aged PCL Blends

The thermal properties of the PCL blends were studied after the samples were subjected to artificial ageing using the xenon lamp to investigate the stability and degradability of the prepared samples. Changes in the structure and chemical composition of the material and the reduction of its molecular mass can result in changes of the functional properties of the material [13,37,55,56,57,58]. The prepared samples were aged in three periods: one, two and three weeks. 

Table 6 shows the selected thermal parameters. It can be seen that the ageing process causes the decrease in crystallization temperature (*T_c_*) for all observed periods but also affects the enthalpy of crystallization (Δ*H_c_*) of neat PCL. The peak melting temperature (*T_m_*) and melting enthalpy (Δ*H_m_*) values did not show significant changes. The degree of crystallinity (*X_c_*) is higher in the second week of ageing but decreases after the third week of exposure. PCL samples with addition of coconut fibers show a change of crystallization peak to slightly higher temperatures and an increase in the degree of crystallinity (*X_c_*) after the first and second week of ageing for 100/0/0.5 samples and a decrease in *X_c_* for 100/0/3.0 samples. After three weeks of exposure, the crystallinity was increased, which is probably due to the effect of chemi-crystallization. Similar results have been previously published where the PCL polymer was exposed to UV-B radiation for nine weeks [59].

Observing Table 6, it can be seen that the melting peak temperature (*T_m_*) and crystallization temperature (*T_c_*) of the unaged and aged PCL/PLA/CF blends showed no significant changes. The enthalpy of fusion (Δ*H_m_*) increased during the exposure periods for the samples without coconut fibers. The results of the thermal properties of PCL/PLA/CF blends show that the addition of coconut fibers causes the decrease in cold crystallization temperature (*T_cc_*) of PLA. Exposure to the environmental chamber decreased the *T_cc_* for all samples, especially after the first week of exposure. This fact, together with the slight increase in the degree of crystallinity of PLA after the exposure periods, suggests that the presence of natural fibers favors the crystallization of PLA by acting as nucleating agents. Similar results have been published using the natural fibers as fillers in various biocomposites [37,60].

**Figure 5:**
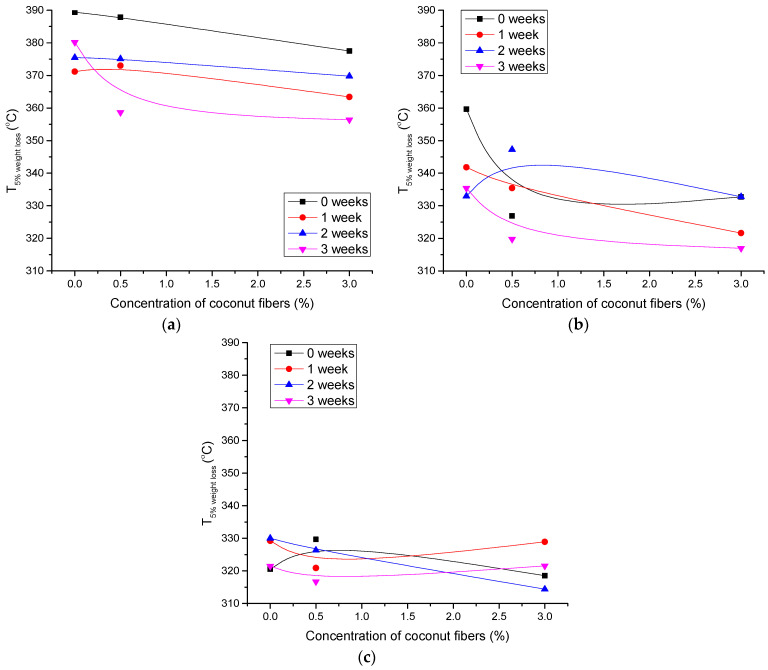
5% weight loss temperature for polymer blends as a function of coconut fiber concentration: (**a**) PCL-0, (**b**) PCL/PLA 80/20 and (**c**) PCL/PLA 70/30.

**Figure 6:**
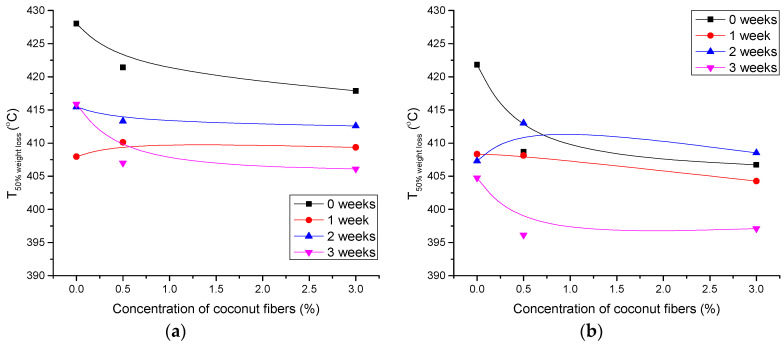

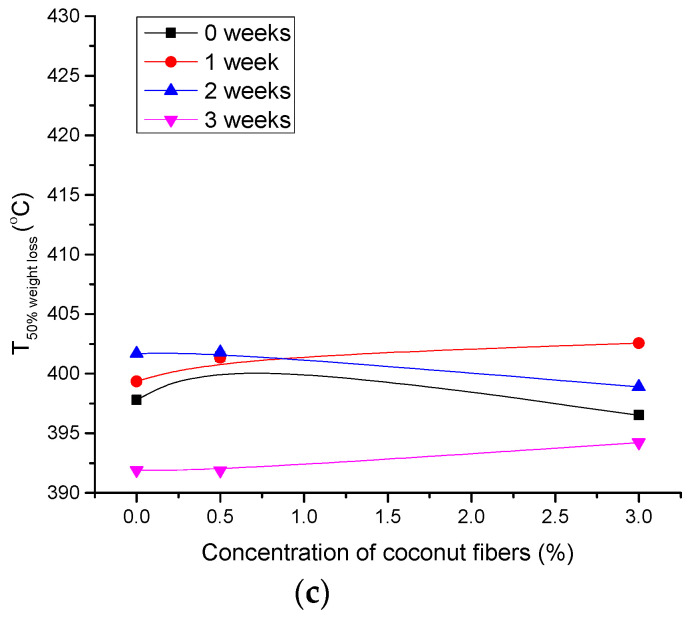
50% weight loss temperature for polymer blends as a function of coconut fiber concentration: (**a**) PCL-0, (**b**) PCL/PLA 80/20 and (**c**) PCL/PLA 70/30.

**Figure 7:**
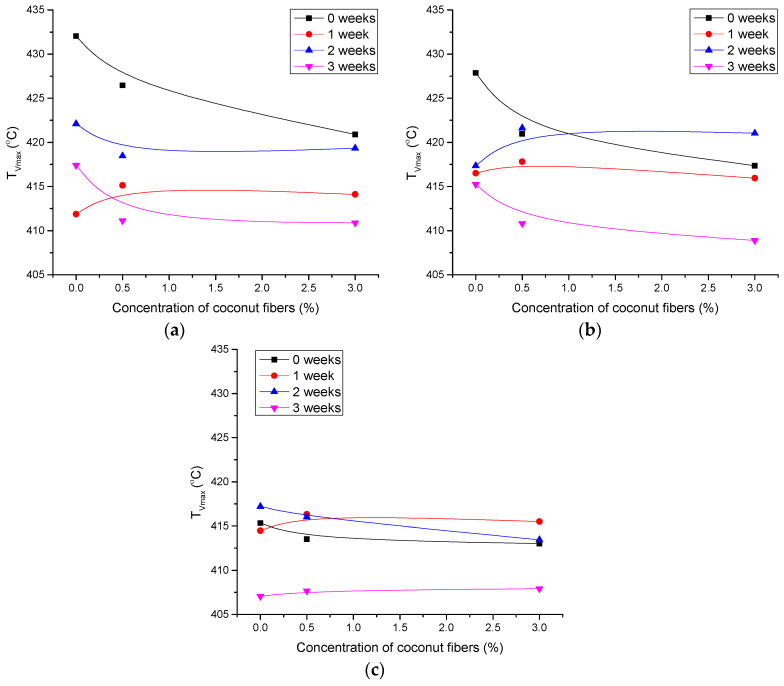
Temperature of maximum degradation rate for polymer blends as a function of CF concentration: (**a**) PCL-0, (**b**) PCL/PLA 80/20 and (**c**) PCL/PLA 70/30.

TGA was performed to determine possible changes in thermal stability and thermal degradation rate of photopolymer blends due to artificial ageing. The results for specific temperatures during the thermal degradation are shown in Figure 5, Figure 6 and Figure 7.

Figure 5 shows the temperatures at which a 5% weight loss occurs in aged polymer blends with the addition of CF. It can be seen that the addition of PLA to the blend causes the temperature to shift to lower values. Specifically, unaged neat PCL without the addition of CF loses 5% weight at 390 °C (Figure 5a), while unaged PCL/PLA/CF 70/30/0 loses 5% weight at 320 °C (Figure 5c). The addition of CF also generally results in the lower degradation onset temperatures for unaged and aged samples. 

The ageing process significantly decreases the thermal stability of the PCL-0 and PCL/PLA 80/20 blends (Figure 5a,b). Similar results are available for the temperatures at 50% weight loss (Figure 6a–c). Ageing for 3 weeks causes a significant shift in temperatures to lower values, especially for PCL-0 and PCL/PLA 80/20 blends. However, higher content of PLA and CF in the blend slightly increases the ageing resistance of the blend (Figure 6c). This can be attributed to the increased crystallinity of PLA after ageing, which was favored by the addition of CF, in agreement with the DSC results (Table 6).

The temperatures at the maximum rate of degradation of the blends are shown in Figure 7. The trend of changes is similar to the temperatures at which 5% and 50% weight loss occurs. 

It can be seen that both the addition of CF to the blends and the ageing process shift the temperatures to lower values. However, the influence of the ageing is more pronounced than the influence of CF. Again, the PCL/PLA 70/30 blend is the most resistant to degradation after the ageing up to 2 weeks. From the obtained results, it can be concluded that CF causes the onset of the thermal degradation of PCL/PLA blends at lower temperatures but at the same time when added in higher concentration (3%) to the blend with increased PLA content leads to stabilization of the thermal resistance of the blend after prolonged ageing.

#### 3.4.2. Mechanical Properties of UV Aged PCL Blends

**Figure 8:**
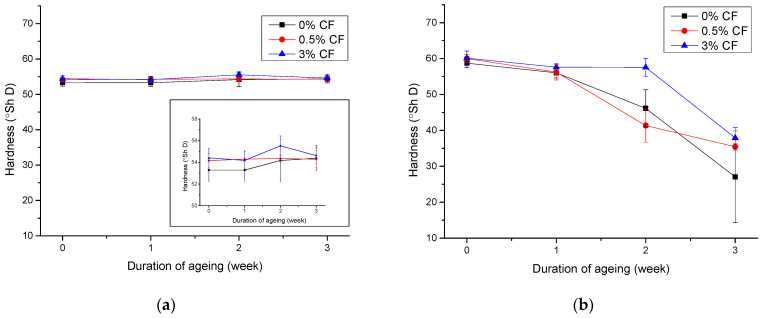
Hardness of aged PCL/PCL/CF blends: (**a**) 100/0/0, 100/0/0.5, 100/0/3.0 and (**b**) 70/30/0, 70/30/0.5, 70/30/3.0.

The results of hardness testing of aged PCL and PCL blends are shown in Figure 8. It is shown in Figure 5 that the addition of CF and PLA to PCL ensures the higher values of hardness. From Figure 8a, it can be seen that the ageing process does not cause significant changes in the hardness of neat PCL and PCL blend with CF. In a detailed preview, it can be seen that after the first week of ageing, all samples had the same hardness value as unaged samples. After two weeks of ageing the hardness increased for about 2° Sh D, but after the third week of ageing the hardness decreased for all samples—the sample with 3% CF added had the least change. Figure 8b shows the changes in hardness for the blend of PCL/PLA 70/30 with addition of CF. It can be seen that the samples without and with the addition of 0.5% fibers had a significant decrease in of hardness after the second week of exposure. These samples became extremely brittle after the second week of exposure, they were cracked and it was not possible to perform the measurement on them. Only the sample with the addition of 3% CF could be measured after the second week of ageing, and its hardness was about 57.6° Sh D, which is a relatively small decrease (the unaged sample has a hardness of 60.1° Sh D). After the third week of ageing, this sample also became too brittle and could not be measured. From these results, it can be concluded that the addition of coconut fiber allows for better stability of the one- and two-week aged samples especially with the addition of 3% CF.

#### 3.4.3. Results of the Surface and Structural Properties of Aged PCL Blends

**Figure 9:**
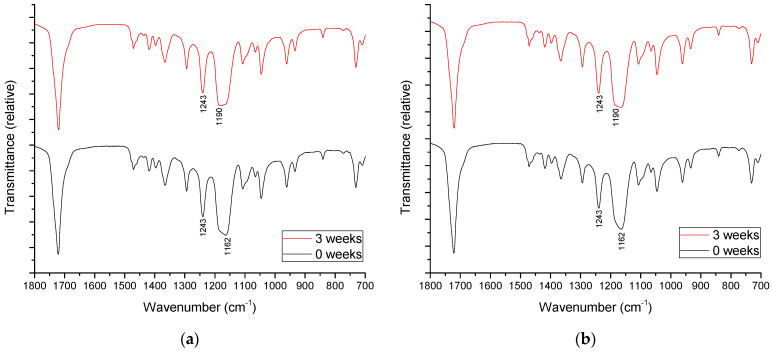
FTIR-ATR spectra of aged PCL/PLA/CF blends: (**a**) 100/0/0 and (**b**) 100/0/3.0.

**Figure 10:**
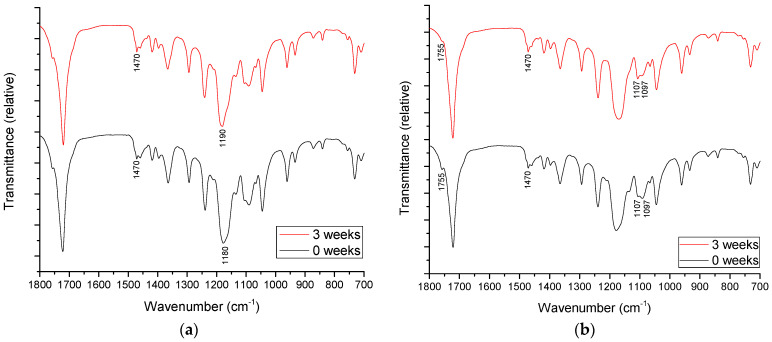
FTIR-ATR spectra of aged PCL/PLA/CF blends: (**a**) 70/30/0 and (**b**) 70/30/3.0.

Figure 9 and Figure 10 show the FTIR-ATR spectra of the selected unaged blends and the blends after 3 weeks of ageing. The results of the FTIR-ATR analysis show the fine specific changes in the surface structure of the materials, which can be related to the changes in surface polarity and crystallinity after the ageing process.

Figure 9a shows the change in the FTIR-ATR spectra of neat PCL after 3 weeks of ageing. The peaks between 1100 and 1400 cm^−1^ are standard peaks for PCL and are related to C–C and C–O stretching and bending. The broad peak at 1162 cm^−1^ which can be assigned to OC–O stretching [61] was shifted to 1190 cm^−1^ after 3 weeks of ageing—a peak that can be assigned to C–O–C stretching in amorphous PCL [62]. When CF is added to neat PCL (Figure 9b), the ageing process causes a change in relative intensity between this peak and the one at 1243 cm^−1^. 

The peak at 1243 cm^−1^ is related to the asymmetric C–O–C stretching in the semi-crystalline state [63]. Therefore, the changes in the FTIR-ATR spectra are indicative of the changes in the crystallinity of the blends detected in the DSC analysis.

When PLA and CF are added to PCL (Figure 10), the changes in the FTIR-ATR spectra after ageing are more pronounced. Without the addition of CF to the PCL/PLA blend, the ageing process causes the shift of the peak which is assigned to C–O–C stretching to 1190 cm^−1^, from 1180 cm^−1^ (Figure 10a). Moreover, the peak at 1470 cm^−1^, which is related to the C–O–C stretching in the semicrystalline phase [63], is increased in intensity after 3 weeks of ageing.

Upon addition of CF to the PCL/PLA 70/30 blend (Figure 10b), a change in crystallinity after ageing is observed in the peak at 1107 cm^−1^. Before ageing, the peak was at 1097 cm^−1^, which can be assigned to the amorphous state of PCL. Its shift to 1107 cm^−1^ can be associated with the increased crystallinity of the blend [63] compared to the unaged sample, which is in agreement with the DSC analysis. The peak at 1470 cm^−1^ undergoes a similar change as in Figure 10a. 

**Figure 11:**
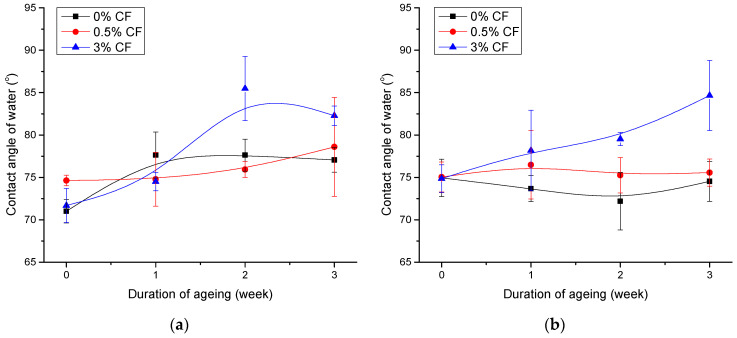
Contact angle of water on aged PCL/PLA/CF blends with addition of coconut fibers: (**a**) 100/0 and (**b**) 70/30.

Moreover, the characteristic peak of PLA at 1755 cm^−1^ in Figure 10b, which is associated with the carbonyl group [64], is decreased in intensity after ageing. This corresponds to the decreased polarity of the surface and the significant increase in the contact angle of water on PCL/PLA/CF blend after ageing, shown in Figure 11. The contact angles of water on aged PCL/PLA/CF blends are shown in Figure 11a,b. The obtained results can be used to analyze the changes in polarity of the aged blends.

In Figure 11a, the increase in the contact angle of water after the ageing process of neat PCL with the addition of CF is most obvious for maximum concentration of CF (3%). In general, CF causes the decrease in surface polarity after the ageing process of the blends. This is consistent with other results of this research, as CF has been shown to accelerate the degradation of PCL and cause changes in crystallinity that could affect the surface properties of the material [35]. Furthermore, it is important to emphasize that the contact angle of water measured on pure coconut fibers was 110.39 ± 4.08°. The increased contact angle of water on the polymer blends with highest CF concentration (3%) can therefore also be attributed to the low polarity of the CFs.

A similar interpretation can be attributed to Figure 11b. Higher concentration of CF caused the explicit increase in the contact angle of water (and thus the decreased surface polarity) after 3 weeks of artificial ageing of the PCL/PLA 70/30 blend. This was caused by the loss of oxygen containing groups from the surface layer of the material, as shown by the FTIR-ATR analysis as well as changes in the crystallinity of PCL and PLA. 

### 3.5. Results of the Image Analysis of PCL/PLA/CF Blends

**Figure 12:**
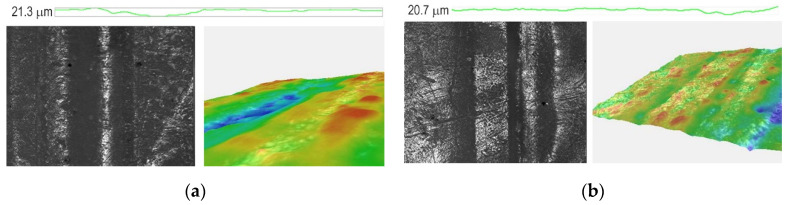
2D and 3D images and a profile of the PCL/PLA/CF blends: (**a**) 100/0/3 and (**b**) 80/20/3 (magnification 40×).

**Figure 13:**
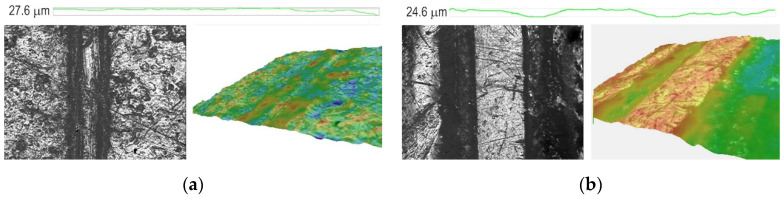
2D and 3D images and a profile of the PCL/PLA/CF blends: (**a**) 70/30/0 and (**b**) 70/30/3 (magnification 40×).

The 2D and 3D images and profiles of selected engraved samples are shown in Figure 12 and Figure 13. Figure 12 shows the images of engraved lines and the profile of PCL/PLA/CF blends, 100/0/3.0 and 80/20/3.0. In Figure 12a, the line is clearly seen on the 2D image, but the profile and 3D image show that the relief and the border of the line are not correctly formed. The depth of engraved line is 21.3 μm. The PCL/PLA/CF 80/20/3 sample shows a series of correctly formed lines in the 2D image and a relatively correctly formed 3D image (Figure 12b). The depth of this engraved line is 20.7 μm. It is possible to engrave plates made from PCL/PLA/CF 100/0/3 and 80/20/3 blends, but apparently due to the higher PCL content and its low melting temperature (60–65 °C), the laser causes partial melting of the PCL, preventing the formation of straight lines with sharp edges [36].

Figure 13 shows the images of PCL/PLA/CF blends, 70/30/0 and 70/30/3.0. It can be seen that the addition of coconut fibers clearly affects the line structure and relief formation in the sample. The 2D image shows a correctly formed line with sharp edges, especially when coconut fiber is added. Apparently, a higher content of PLA, the melting temperature of which is higher than that of PCL (about 160 °C), affects the material in such a way that it is possible to engrave it and obtain appropriate line shapes. The depths of those engraved lines are 27.6 and 24.6 μm. It can be concluded that addition of coconut fibers has a positive effect on the PCL/PLA blends and makes it possible to obtain a more stable material, more suitable for laser engraving and for the relief printing plate application.

After the surface and thermal analysis of the prepared PCL/PLA/CF blends, as well as the performed hardness measurements, FTIR-ATR spectroscopy and microscopy, it can be concluded that the formulations of the material with higher PLA and CF concentrations are highly applicable in the production of the relief printing plates, especially for the embossing processes. The detailed analysis of the newly produced biocompatible and biodegradable materials’ mechanical properties is an important and interesting research area [65,66]. Therefore, future research will explore the application of the new fillers/compatibilizers in the PCL/PLA blends as well as analyze and optimize their mechanical and other properties to expand and improve their potential for applicability in relief printing processes.

## 4. Conclusions

In this study, three-component blends containing PCL, PLA and coconut fibers (CFs) in different proportions were prepared as a potential material for production of printing plates for embossing process. Thermal, mechanical and surface analyses of PCL/PLA/CF blends were carried out, and the influence of artificial ageing of the prepared blends was observed. 

The results of DSC data for the cooling and second heating cycles of the unaged blends showed that the addition of PLA polymer has a significant effect on reducing the degree of crystallinity of PCL in PCL/PLA blends. The addition of CF to PCL/PLA 80/20 blends resulted in a significant increase in the degree of crystallinity of PCL. Results of TG and DTG measurements have shown that CF accelerates the thermal degradation of PCL/PLA blends. The hardness test proved that the addition of CF increases the hardness of all samples. From the results of interfacial interactions and adhesion parameters in PCL/PLA/CF blends, it can be concluded that CF is incorporated into the PLA matrix and not at the interface of PCL and PLA. 

After analyzing the thermal properties of ageing blends, it can be concluded that ageing process caused the decrease in crystallization temperature for all the observed periods but also affected the enthalpy of fusion of neat PCL samples. Obtained results suggest that the presence of natural fibers favors the crystallization of PLA, as they act as nucleating agents. 

The results obtained from the TG and DTG curves showed that the higher content of PLA and CF in the blend slightly increased the ageing resistance of the blend. The results showed that CF cause the onset of thermal degradation of PCL/PLA blends at lower temperatures; at the same time, the addition at higher content (3%) to the blend with increased PLA content leads to stabilization of the thermal resistance of the blend after prolonged ageing.

The results of hardness of aged samples without addition of fibers and with addition of 0.5% CF showed significant change in hardness and brittleness due to the ageing process. 

The results of the FTIR-ATR analysis of the aged blends showed that the changes in the surface structure can be related to the changes in the surface polarity and crystallinity after the ageing process. 

The 2D and 3D images and profiles of PCL/PLA/CF blends showed that higher content of PLA and the addition of CF resulted in the formation of a more suitable material for the production of relief printing plates.

## Figures and Tables

**Table 1:** Properties of PCL and PLA.

Material	Physical Properties			Mechanical Properties	
	Density, *ρ* (g·cm^−3^)	*T_m_* (°C)	*T_g_* (°C)	Tensile Strength (MPa)	Tensile Elongation (%)
PCL	1.145	58–60	−60	20	800
PLA	1.24	150–160	55–60	60	3.5

**Table 2:** Sample names and blend composition.

Sample Name	Ratio (%)	Sample Name	Ratio (%)
PCL/PLA/CF	100/0/0	PCL/PLA/CF	80/20/3.0
PCL/PLA/CF	100/0/0.5	PCL/PLA/CF	70/30/0
PCL/PLA/CF	100/0/3.0	PCL/PLA/CF	70/30/0.5
PCL/PLA/CF	80/20/0	PCL/PLA/CF	70/30/3.0
PCL/PLA/CF	80/20/0.5		

**Table 3:** DSC data for the cooling and second heating cycle of PCL/PLA/CF blends.

PCL/PLA/CF (wt%)	PCL						PLA				
	*T_g_* (°C)	*T_m_* (°C)	Δ*H_m_* (J·g^−1^)	*T_c_* (°C)	Δ*H_c_* (J·g^−1^)	*X_c_* _(PCL)_ (%)	*T_cc_* (°C)	Δ*H_cc_* (J·g^−1^)	*T_m_* (°C)	Δ*H_m_* (J·g^−1^)	*X_c_*_(PLA)_ (%)
100/0/0	−63.6	56.3	74.0	32.9	70.1	53.1	-	-	-	-	-
100/0/0.5	−64.5	55.7	73.4	26.5	64.5	52.7	-	-	-	-	-
100/0/3.0	−63.9	56.2	77.0	27.8	64.8	55.2	-	-	-	-	-
80/20/0	−63.7	57.4	48.8	32.2	47.2	43.8	98.1	4.6	168.2	8.1	17.1
80/20/0.5	−63.9	56.3	65.3	33.2	60.0	58.6	94.2	3.3	166.8	9.5	29.3
80/20/3.0	−64.5	56.3	65.9	33.2	58.2	59.2	94.1	5.1	166.8	9.9	23.0
70/30/0	−63.0	56.4	46.2	31.1	40.9	47.4	99.5	8.9	167.6	12.5	11.4
70/30/0.5	−63.2	56.6	46.0	31.2	38.9	47.2	95.9	6.9	167.3	12.4	17.2
70/30/3.0	−64.0	56.4	37.7	30.7	39.9	38.3	93.8	5.8	166.4	12.4	20.8

**Table 4:** Hardness of PCL/PCL/CF blends.

PCL/PLA/CF (wt%)	° Sh D	SD
100/0/0	53.3	1.0
100/0/0.5	54.2	0.7
100/0/3.0	54.4	0.9
80/20/0	55.0	1.1
80/20/0.5	58.7	1.3
80/20/3.0	58.9	1.4
70/30/0	58.7	1.3
70/30/0.5	60.0	1.1
70/30/3.0	60.1	1.9

**Table 5:** Surface free energy and adhesion parameters in PCL/PLA/CF blends.

SFE	*γ^total^*	*γ^D^*	*γ^P^*	Adhesion Parameters	*γ*_mf_	*W*_mf_	*S*_mf_
CF	33.1	31.9	1.1	PLA/CF	1.4	77.4	11.3
PCL	36.8	30.3	6.4	PCL/CF	2.2	67.6	1.5
PLA	45.7	39.8	7.7	PCL/PLA	0.5	82.0	−9.5

**Table 6:** DSC data for the cooling and second heating cycle of PCL/PCL/CF blends exposed to radiation in an environmental chamber for up to 3 weeks.

Ageing (Weeks)	PCL/PLA/CF (wt%)	PCL						PLA				
		*T_g_* (°C)	*T_m_* (°C)	Δ*H_m_* (J·g^−1^)	*T_c_* (°C)	Δ*H_c_* (J·g^−1^)	*X_c_*(PCL) (%)	*T_cc_* (°C)	Δ*H_cc_* (J·g^−1^)	*T_m_* (°C)	Δ*H_m_* (J·g^−1^)	*X*_*c*(PLA)_ (%)
0	100/0/0	−63.6	56.3	74.0		70.1	53.1	-	-	-	-	-
1		−64.9	56.2	75.5	28.6	69.6	54.2					
2		−63.8	55.3	80.6	30.5	76.5	57.9					
3		−64.5	55.4	75.6	29.6	73.3	54.3					
0	100/0/0.5	−64.5	55.7	73.4	26.5	64.5	52.7	-	-	-	-	-
1		−64.1	56.0	77.4	27.6	65.9	55.6					
2		−64.1	55.9	78.0	29.3	67.2	56.0					
3		−63.2	56.5	79.7	29.8	68.9	57.0					
0	100/0/3.0	−63.9	56.2	77.0	27.8	64.8	55.2	-	-	-	-	-
1		−65.1	56.0	76.3	29.2	66.3	54.8					
2		−63.6	57.0	73.8	30.4	63.8	53.0					
3		−64.8	55.8	78.4	30.8	69.5	56.3					
0	80/20/0	−63.7	57.4	48.8	32.2	47.2	43.8	98.1	4.6	168.2	8.1	16.1
1		−63.8	55.5	59.9	33.6	58.7	53.8	90.9	4.5	166.0	9.7	24.5
2		−63.7	56.4	58.8	33.2	56.0	52.8	92.9	5.1	166.7	9.5	20.9
3		−63.4	55.1	61.4	33.6	61.4	55.1	90.9	4.8	166.1	9.2	20.7
0	80/20/0.5	−63.9	56.3	65.3	33.2	60.0	58.6	94.2	3.3	166.8	9.5	29.3
1		−62.6	55.5	64.9	33.6	60.2	58.3	90.4	4.0	165.8	9.5	25.8
2		−63.4	56.6	62.1	34.1	58.7	55.7	92.5	2.7	167.3	7.5	22.9
3		−61.2	55.5	56.9	33.4	61.7	51.0	89.7	1.4	165.4	8.5	33.2
0	80/20/3.0	−64.5	56.3	65.9	33.2	58.2	59.2	94.1	5.1	166.8	9.9	22.8
1		−64.8	55.9	56.2	33.1	56.4	50.4	90.4	4.6	165.5	9.8	24.5
2		−63.9	57.0	53.9	33.5	51.8	48.4	93.2	4.5	166.8	9.4	23.4
3		−64.6	55.6	56.4	32.8	55.7	50.6	89.2	3.7	166.0	9.1	25.1
0	70/30/0	−63.0	56.4	46.2	31.1	40.9	47.4	99.5	8.9	167.6	12.5	11.4
1		−63.6	55.7	55.9	33.6	53.3	57.3	95.8	9.1	166.5	14.1	15.4
2		−63.2	55.1	55.8	33.5	57.5	57.2	96.2	9.4	166.1	12.2	8.9
3		−63.6	55.9	55.7	33.9	52.9	57.2	98.4	9.9	166.9	12.9	9.7
0	70/30/0.5	−63.2	56.6	46.0	31.2	38.9	47.2	95.8	6.9	167.3	12.4	17.2
1		−64.0	55.1	55.6	33.5	55.5	57.0	90.6	3.7	165.5	12.3	27.2
2		−63.4	55.6	54.4	33.6	53.9	55.8	92.4	4.6	166.1	13.6	28.4
3		−63.0	55.7	55.2	33.9	55.2	56.6	92.9	4.8	166.1	13.9	28.6
0	70/30/3.0	−64.0	56.4	37.7	30.7	39.2	38.3	93.8	5.8	166.4	12.4	20.8
1		−65.3	55.8	47.4	32.4	49.8	48.6	91.4	5.1	165.8	13.3	26.0
2		−65.1	55.6	51.4	33.3	50.5	52.7	92.4	5.6	165.8	13.6	25.2
3		−63.4	56.4	48.8	32.7	48.6	50.0	92.1	4.0	166.2	12.7	27.6

## Data Availability

Not applicable.
